# Co/CoP Nanoparticles Encapsulated Within N, P-Doped Carbon Nanotubes on Nanoporous Metal-Organic Framework Nanosheets for Oxygen Reduction and Oxygen Evolution Reactions

**DOI:** 10.1186/s11671-020-03316-x

**Published:** 2020-04-15

**Authors:** Xinxin Yang, Hongwei Mi, Xiangzhong Ren, Peixin Zhang, Yongliang Li

**Affiliations:** 1 College of Chemistry and Environmental Engineering, Shenzhen University, Shenzhen, 518060 Guangdong People’s Republic of China; 2 Guangdong Flexible Wearable Energy Tools Engineering Technology Research Centre, Shenzhen University, Shenzhen, 518060 Guangdong People’s Republic of China

**Keywords:** Transition metal phosphide, Metal-organic frameworks, Bifunctional electrocatalyst

## Abstract

Herein, Co/CoP nanoparticles encapsulated with N, P-doped carbon nanotubes derived from the atomic layer deposited hexagonal metal-organic frameworks (MOFs) are obtained by calcinations and subsequent phosphating and are employed as electrocatalyst. The electrocatalytic performance evaluations show that the as-prepared electrocatalyst exhibits an overpotential of 342 mV at current density of 10 mA cm^−2^ and the Tafel slope of 74 mV dec^−1^ for oxygen evolution reaction (OER), which is superior to the most advanced ruthenium oxide electrocatalyst. The electrocatalyst also shows better stability than the benchmark RuO_2_. After 9 h, the current density is only decreased by 10%, which is far less than the loss of RuO_2_. Moreover, its onset potential for oxygen reduction reaction (ORR) is 0.93 V and follows the ideal 4-electron approach. After the stability test, the current density of the electrocatalyst retains 94% of the initial value, which is better than Pt/C. The above results indicate that the electrocatalyst has bifunctional activity and excellent stability both for OER and ORR. It is believed that this strategy provides guidance for the synthesis of cobalt phosphide/carbon-based electrocatalysts.

## Introduction

The development of modern society depends on energy supply to a large extent, but with environmental problems induced by the burning of fossil fuels and the aggravation of energy shortage, it is necessary to find new conversion systems or renewable energy [1–4]. Fuel cells and metal-air batteries are considered to be promising energy systems; however, their poor energy conversion efficiency and short life span are the main bottlenecks that limit their widespread use [5–9]. These deficiencies are primarily on account of the inherent sluggish kinetics of the oxygen evolution reaction (OER) and oxygen reduction reaction (ORR) [10–13]. Especially, OER plays a very important role in metal-air batteries and water splitting. However, the slow kinetics of it usually result in low reaction rates and high electrode overpotential, hindering the development such energy systems. Currently, the most accepted theory explains the OER process under alkaline conditions is as follows:

1 $$ \mathrm{M}+\mathrm{OH}\to \mathrm{M}-\mathrm{OH}+{\mathrm{e}}^{-} $$

2 $$ \mathrm{M}-\mathrm{OH}+{\mathrm{O}\mathrm{H}}^{-}\to \mathrm{M}-{\mathrm{O}}^{-}+{\mathrm{H}}_2\mathrm{O} $$

3 $$ \mathrm{M}-{\mathrm{O}}^{-}\to \mathrm{M}-\mathrm{O}+{\mathrm{e}}^{-} $$

4 $$ 2\mathrm{M}-\mathrm{O}\to 2\mathrm{M}+{\mathrm{O}}_2 $$

ORR as a cathode reaction of a fuel cell is a key factor restricting the efficiency of the cell. During the reaction, a variety of intermediate oxygen species are generated, and the reaction process is relatively complicated. Under alkaline conditions, there are two reaction modes:

2e^−^ path:

5 $$ {\mathrm{O}}_2+{\mathrm{H}}_2\mathrm{O}+2{\mathrm{e}}^{-}\to \mathrm{H}{\mathrm{O}}_2+\mathrm{O}{\mathrm{H}}_2^{-} $$

6 $$ \mathrm{H}{\mathrm{O}}_2^{-}+\mathrm{O}{\mathrm{H}}^{-}+2{\mathrm{e}}^{-}\to 3\mathrm{O}{\mathrm{H}}^{-}\ \mathrm{or}\ \mathrm{H}{\mathrm{O}}_2^{-}+\mathrm{O}{\mathrm{H}}^{-}+2{\mathrm{e}}^{-}\to 3\mathrm{O}{\mathrm{H}}^{-} $$

4e^−^ path:

7 $$ {\mathrm{O}}_2+2{\mathrm{H}}_2\mathrm{O}+4{\mathrm{e}}^{-}\to 4\mathrm{O}{\mathrm{H}}^{-} $$

Therefore, the exploration of inexpensive, highly efficient, and durable electrocatalysts is necessary to promote the practical application of these renewable resources [14, 15]. At present, precious metals are considered to be the most active electrocatalysts such as Pt, Ru, Ir, and their alloys, but high cost, scarcity, and lack of bifunctional catalysis have seriously hindered their commercialization [16–20]. Therefore, the pursuit of bifunctional, stable and inexpensive electrocatalysts is urgently needed for the demand of commercialization. Transition metal phosphides (TMPs) are promising alternative candidates of which Co^2+^ in Co_x_P_y_ provides OH^−^ adsorption center and converts it into products, while negative P center accelerates OH^−^ adsorption to Co^2+^, resulting in low cost, excellent performance, high efficiency, and good durability [21–24]. Many researchers have made great efforts to design CoP_x_ nanostructures with diverse and high electrocatalytic activity. Since the activity depends largely on their surface properties, many research focused on the structure engineering of electrocatalysts to expose catalytic active sites as much as possible, for example various nanostructured TMPs, including nanoparticles [25, 26], nanowires [27, 28], nanotubes [29, 30], and nanorods [31, 32] are developed and most of them showed good electrocatalytic performance. There have been many reports that high-efficiency and strong cobalt-based materials were considered as a promising OER electrocatalyst due to its high efficiency, high abundance, and good stability in recent years. Cobalt phosphide (CoP) is one of the TMPs families. Due to the novel characteristics of CoP, its application in battery electrocatalysis and photocatalysis has received extensive attention. It provides a large number of active sites for electrochemical reaction to promote electrocatalytic activity [33]. CoP not only solves the problems of insufficient reserves, high price, and poor stability of Ru and Ir electrocatalysts, but also has good catalytic performance for OER [34, 35]. In addition, CoP has neutral alkali resistance and is advantageous for electrochemical stability. However, the conductivity of CoP is poor, which seriously affects its electrocatalytic activity [36].

Metal-organic frameworks (MOFs) are a series of adjustable organic-inorganic hybrid materials with adjustable structures [37, 38]. In short, metal ions are uniformly dispersed at the atomic level in the MOFs precursor, and the presence of organic ligands in the MOFs enables them to be calcined into various carbon materials without introducing an external carbon source [39]. As a general precursor for the preparation of TMPs, MOFs compounds have been extensively studied by reason of their large specific surface area, high porosity, and structural coordination [40, 41]. In general, MOFs carbonization process requires high temperature calcination, which will damage the original MOFs structure and cause agglomeration of the metal center [42]. Direct use of MOFs as an electrocatalyst can utilize its good structure, but their stability is relatively low, and catalytic activity is poor, especially under strong alkaline and acidic solution conditions [43, 44]. If reasonably designed, the hybrid electrocatalyst combining TMPs and MOFs not only enhances the intrinsic catalytic activity but also utilizes the well-defined porous structure of MOFs. More importantly, the center of the coordinated unsaturated metal MOFs is more favorable for adsorption oxygen-containing substances, which will further enhance catalytic performance [45].

Herein, we report the preparation of nanotubes (CNTs) derived from N-doped porous MOFs nanosheets (NPM) via atomic layer deposition (ALD) techniques, with Co/CoP nanoparticles encapsulated at the tip of the nanotubes. The controlled portion of the Co-MOFs creates a Co/CoP species during the phosphating process, resulting in a hybrid nanostructure that have a large specific surface area. The as-prepared product is used as electrocatalyst, exhibiting a bifunctional feature in electrochemical performance for both OER and ORR. Its onset potential was 0.93 V for ORR while the overpotential was about 342 mV with the Tafel slope of 74 mV dec^−1^ for OER. Moreover, the electrocatalyst also showed excellent stability for both reactions.

## Methods

### Materials

Potassium hydroxide (KOH), 2-methylimidazole (C_4_H_6_N_2_), sodium hypophosphite (NaH_2_PO_2_), and zinc nitrate hexahydrate (Zn(NO_3_)_2_·6H_2_O) were purchased from Shanghai Macklin Biochemical Technology Co., Ltd. Cobaltocene ((η5-C_5_H_5_)_2_Co) was purchased from Suzhou Fornano Co., Ltd. All of the above chemicals are analytically pure. Nafion solution (5 wt%) was purchased from Shanghai Hesen Co., Ltd.

### Synthesis of Electrocatalysts

First, 90 mL of deionized water including 0.33 g of zinc nitrite hexahydrate was slowly added to another prepared solution of 90 mL of deionized water including 0.985 g of 2-methylimidazole, then stirred continuously for 24 h at 25 °C. This mixture was centrifuged with ethyl alcohol absolute several times and dried at 70 °C in ambient air, the finally obtained white powder denoted as NPM.

The electrocatalyst (denoted as NPMCNT) was deposited by using equipment of KEMICRO PEALD-200A (Kemin Co. Ltd, China). During the PE-ALD process, Cobaltocene (CoCp_2_) was used as Co source and oxygen plasma (O_2_, 99.999%) was used as O source. This sedimentary process was deposited at 200 °C in a vacuum reaction chamber and argon (Ar, 99.999%) as the carrier gas was used to purge excess sources. The Co source temperature was 100 °C. The second source (oxygen plasma) was maintained at 25 °C. The deposition process consists of 200 cycles and each cycle consists of 4 steps: Co source, Ar, oxygen plasma, and Ar. The dose times of Co source and oxygen plasma were 3 and 20 s respectively, and the Ar purge time was 50 s. The obtained powder was annealed at 925 °C for 2 h under N_2_ with the heating rate of 2 °C min^−1^. The acquired product was named NPMCNT.

The 10 mg NPMCNT electrocatalyst obtained above was placed upstream of the tube furnace, and 300 mg of sodium hypophosphite was placed downstream of the tube furnace, and then annealed at 350 °C for 2 h under N_2_ with the heating rate of 2 °C min^-1^. The acquired product was named NPMCNT-300. The NPMCNT-50, NPMCNT-100, NPMCNT-200, and NPMCNT-400 were prepared using the same procedure but the sodium hypophosphite amount was changed as 50, 100, 200, and 400 mg, respectively.

### Physical Characterization

The crystallite structure was acquired by X-ray powder diffraction (XRD, Empyrean, PANalytical) with Cu Kα radiation. The morphology was confirmed by the field emission scanning electron microscope (FESEM, JSM-7800F). The microstructure was observed by transmission electron microscope (FETEM, JEM-200). The element distribution was measured by energy dispersive X-ray spectroscopy (EDS, JEM-F200). The relationship of the bond energy was collected by X-ray photoelectron spectroscopy (XPS, K-Alpha+). Nitrogen adsorption–desorption isotherms were collected on a BELSORP-max II instrument.

### Electrochemical Measurements

The 5 mg of the NPMCNT-300 electrocatalyst was added into the mixed solution containing 100 μm Nafion (5 wt%, DuPont) and 1 mL ethyl alcohol absolute, then treated with an ultrasound for 30 min to form a well-proportioned mixture. Twelve microliter of the homogeneous mixture was dropped several times onto pre-polished glassy carbon electrode, and then dried it naturally at room temperature.

All the electrochemical measurements were measured by CHI760E workstation (China) with three-electrode system. The ORR and OER activities were investigated using a rotating ring-disk electrode (RRDE, Φ_d_ = 4 mm, Φ_Pt ring_ = inner/outer-ring diameter 5.0/7.0 mm, ALS, Japan) in 0.1 M KOH. The smooth carbon electrode with deposited electrocatalyst, the platinum wire, and the Ag/AgCl electrode were served as working, counter, and reference electrodes, respectively. The linear sweep voltammogram (LSV) technique was used to test the electrochemical catalytic activity with voltage range 1.1653~0.1653 V (vs. RHE), with rotation speed of electrode 1600 rpm and the scan rate of 5 mV s^−1^ in 0.1 M KOH electrolyte. All potential values convert to that of a reversible hydrogen electrode (RHE) by the following formula:

8 $$ {E}_{\mathrm{RHE}}={E}_{\mathrm{Ag}/\mathrm{AgCl}}+0.0591\times \mathrm{pH}+0.197\ \left(\mathrm{V}\right). $$

At different various rotational speeds (400, 625, 900, 1225, 1600, and 2025 rpm), the value of the transfer electron number (*n*) of the LSV curve during the ORR obtained by RDE can be calculated by the following Koutecky-Levich (K-L) equation:

9 $$ \frac{1}{j}=\frac{1}{j_k}+\frac{1}{j_d}=\frac{1}{nFK{C}_{O_2}}+\frac{1}{B{\omega}^{\raisebox{1ex}{$1$}\!\left/ \!\raisebox{-1ex}{$2$}\right.}} $$

10 $$ B=0.2{nFC}_{O_2}{D}_{O_2}^{\raisebox{1ex}{$1$}\!\left/ \!\raisebox{-1ex}{$3$}\right.}{V}^{\raisebox{1ex}{$-1$}\!\left/ \!\raisebox{-1ex}{$6$}\right.} $$

where *j* is the measured current density, *j*_*k*_ is the estimated kinetic limiting current densities, *n* is the overall number of electrons transferred per oxygen molecule. *F* is the Faraday constant (*F* = 96,485 C mol^−1^), and *ω* is the angular velocity of the disk (*ω* = 2π*N, N* is the linear rotation speed), ${C}_{{\mathrm{O}}_2}$ is the bulk concentration of O_2_ in the electrolyte (0.1 M KOH, 1.2 × 10^−6^ mol cm^−3^), ${D}_{{\mathrm{O}}_2}$ is the diffusion coefficient of O_2_ in the electrolyte (1.9 × 10^−5^ cm^2^ s^−1^), *ν* is the kinematic viscosity of the electrolyte (0.01 cm^2^ s^−1^), *k* is the electron transfer rate constant. The constant 0.2 is generally accepted when the rotating speed is presented in rpm. The electron transfer number (*n*) and the yield of H_2_O_2_ tested by the RRDE measurement and calculated by the ring and disk currents by the following formulas:

11 $$ n=4\times \frac{I_{\mathrm{disk}}}{I_{\mathrm{disk}}+{I}_{\mathrm{ring}}/N} $$

12 $$ {\mathrm{HO}}_2^{-}\left(\%\right)=100\times \frac{2{I}_{\mathrm{ring}}/N}{I_{\mathrm{disk}}+{I}_{\mathrm{ring}}/N} $$

where *I*_ring_ and *I*_disk_ are the ring and the disk currents, respectively. *N* value was adjusted to 0.43 using a [Fe(CN)_6_]^4−/3−^ redox couple.

The electrochemical active surface area (ECSA) was measured at various scanning rates (5–35 mV s^−1^) and 0~0.15 V (vs. Ag/AgCl) by cyclic voltammetry (CV) measurement.

## Results and Discussion

### XRD and SEM Characterization

**Fig. 1:**
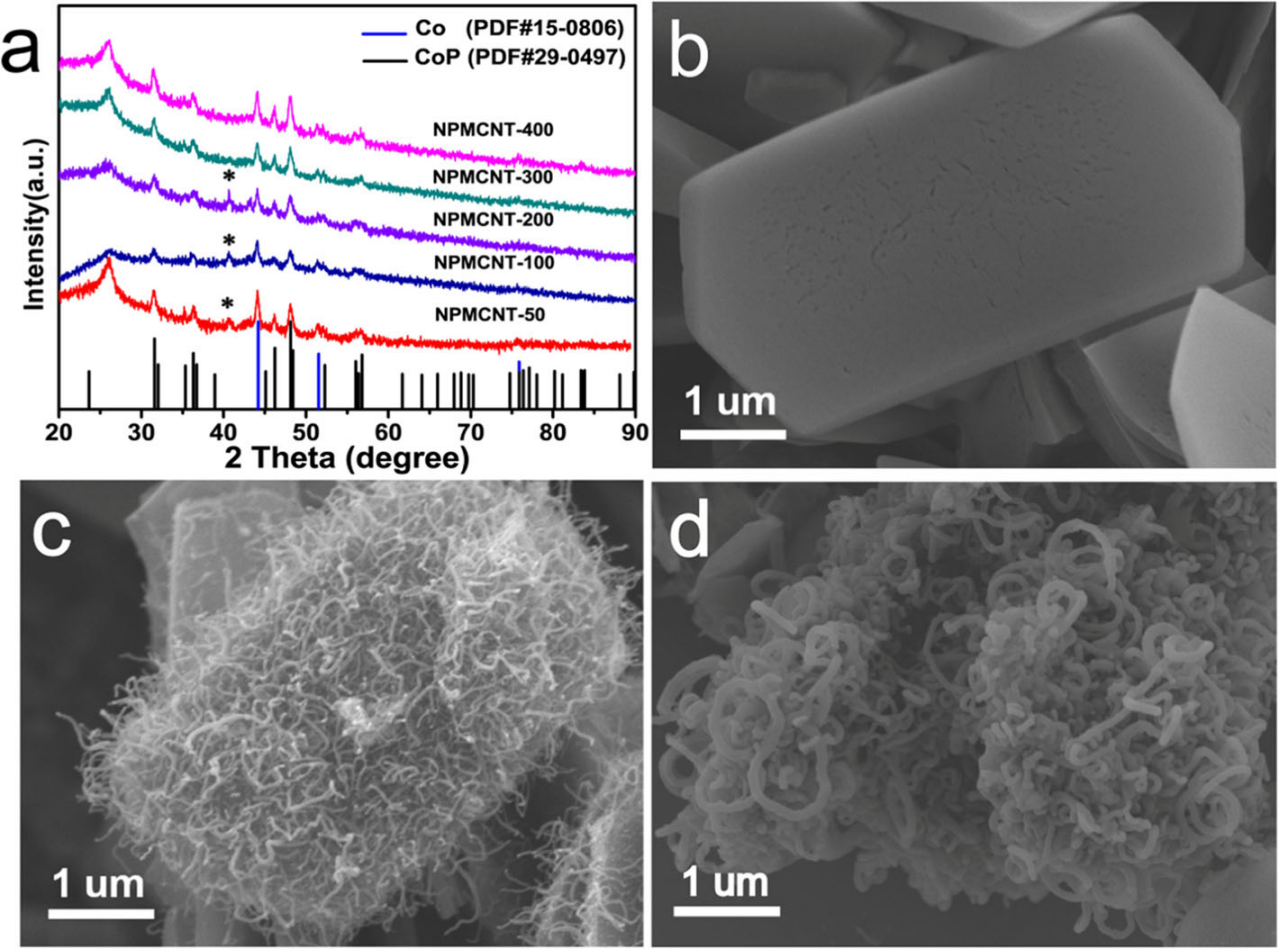
a The XRD patterns. SEM images of **b** NPM, **c** NPMCNT-300, **d** NPMCNT-400

In Fig. 1a, typical patterns of Co (PDF no.15-0806) and CoP (PDF no.29-0497) are shown in the XRD patterns of NPMCNT composites under different phosphorus source intakes. It is worth noting that the intake of different phosphorus sources during phosphating process will lead to the formation of different products. When the phosphorus source was 50, 100, and 200 mg, it was obvious that the characteristic peak of Co_2_P at 40.7° appeared. However, when the phosphorus source intake was increased to 300 and 400 mg, the characteristic peak of Co_2_P was disappeared. Therefore, the Co/CoP hybrid was obtained when using the latter mass of phosphorus source. The characteristic peaks displayed between 20° and 30° are due to the carbon clothes formed after MOFs calcination. It can be observed in Fig. 1b, NPM presents a hexagonal sheet structure after pyrolysis at high temperature and Fig. 1 c shows that CNTs were generated evenly on the surface of NPM sheet. Herein, according to our previous work [1], CoOx was deposited by ALD on the surface of NPM at 200 °C, which is reduced to Co by carbon at 925 °C and nanotubes are grown. When the phosphorus source intake was 400 mg, the nanotubes were already bonded together instead of being individual distribution as shown in Fig. 1d.

### TEM Characterization

**Fig. 2:**
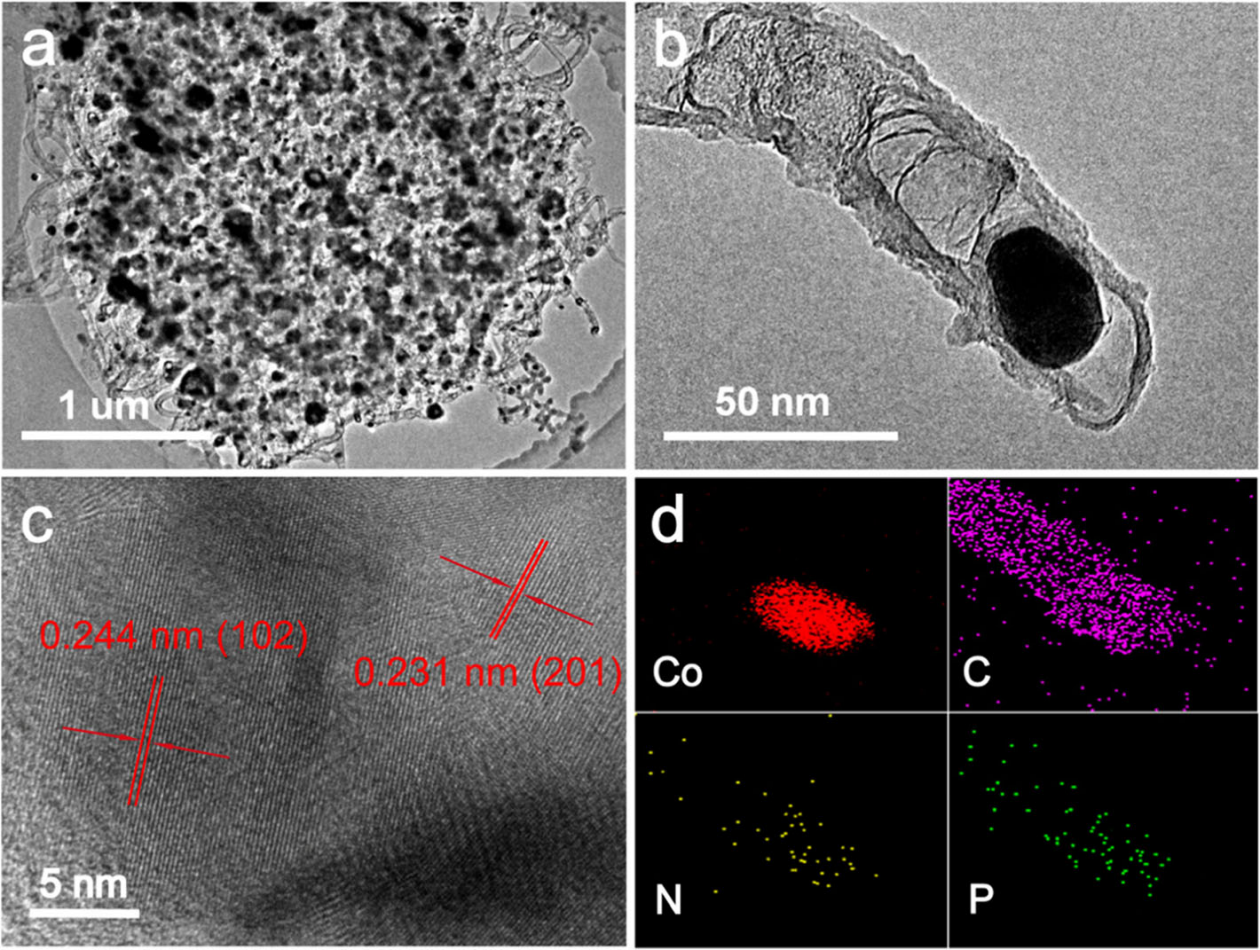
TEM images of the **a** NPMCNT-300 electrocatalyst and **b** CoP nanoparticles encapsulated in the CNT tip derived from the carbon layer. **c** HRTEM image of NPMCNT-300 electrocatalyst. **d** EDS elemental mapping corresponding to the area in TEM image of NPMCNT-300 electrocatalyst

TEM observation shows the entire view of the entire NPMCNT-300. Obviously, the bulk morphology of MOFs was preserved, and a large number of nanotubes were clearly visible at the edges, as well as the nanoparticles are encapsulated in the carbon nanotubes (Fig. 2a). The high-resolution TEM in Fig. 2b further proves the nanoparticles encapsulated at the tip of carbon nanotubes. Co nanoparticles will catalyze the derivation of CNTs from MOFs, which can improve the conductivity of the entire hybrid structure. And few layer of graphitic carbon layer can prevent the embedded Co and CoP nanoparticles from corrosion, aggregation, and oxidation during the electrocatalytic processes, which results in excellent durability and stability in harsh environments. In addition, the N-doped CNTs structure derived from MOFs provides an effective way to adjust the electronic structure of the electrocatalyst, thereby promoting catalytic performance. The plane spacing in Fig. 2c is determined to be 0.244 and 0.231 nm, identifying with the (102) and (201) crystal plane of the CoP nanoparticles, respectively. The EDS analysis (Fig. 2d) further confirmed that the nanoparticles were encapsulated at the tip of the CNTs, and the mapped image also shows that P was not only present in the CoP nanoparticles but also in the CNTs. The N-doped carbon-supported nanomaterials can be obtained from organic monomers (2-methylimidazole) by heat treatment without using any external source. For the phosphorus doping, the NaH_2_PO_2_ is as the phosphorus source and will dope into the carbon structure by heat treatment at 350 °C. In this work, doping of different heteroatoms can modify the chemical structure and electronic structure of the electrocatalyst, so that the surface of the derived nanotubes will have more catalytically active sites. Some reports indicated that carbon defects can generate active sites by adjusting the electronic structure and surface polarity of carbon, thereby improving the electrocatalytic performance. Therefore, carbon-based cobalt phosphide nanocomposites doped with multiple heteroatoms have more excellent electrocatalytic activity [46–48].

### XPS Characterization

**Fig. 3:**
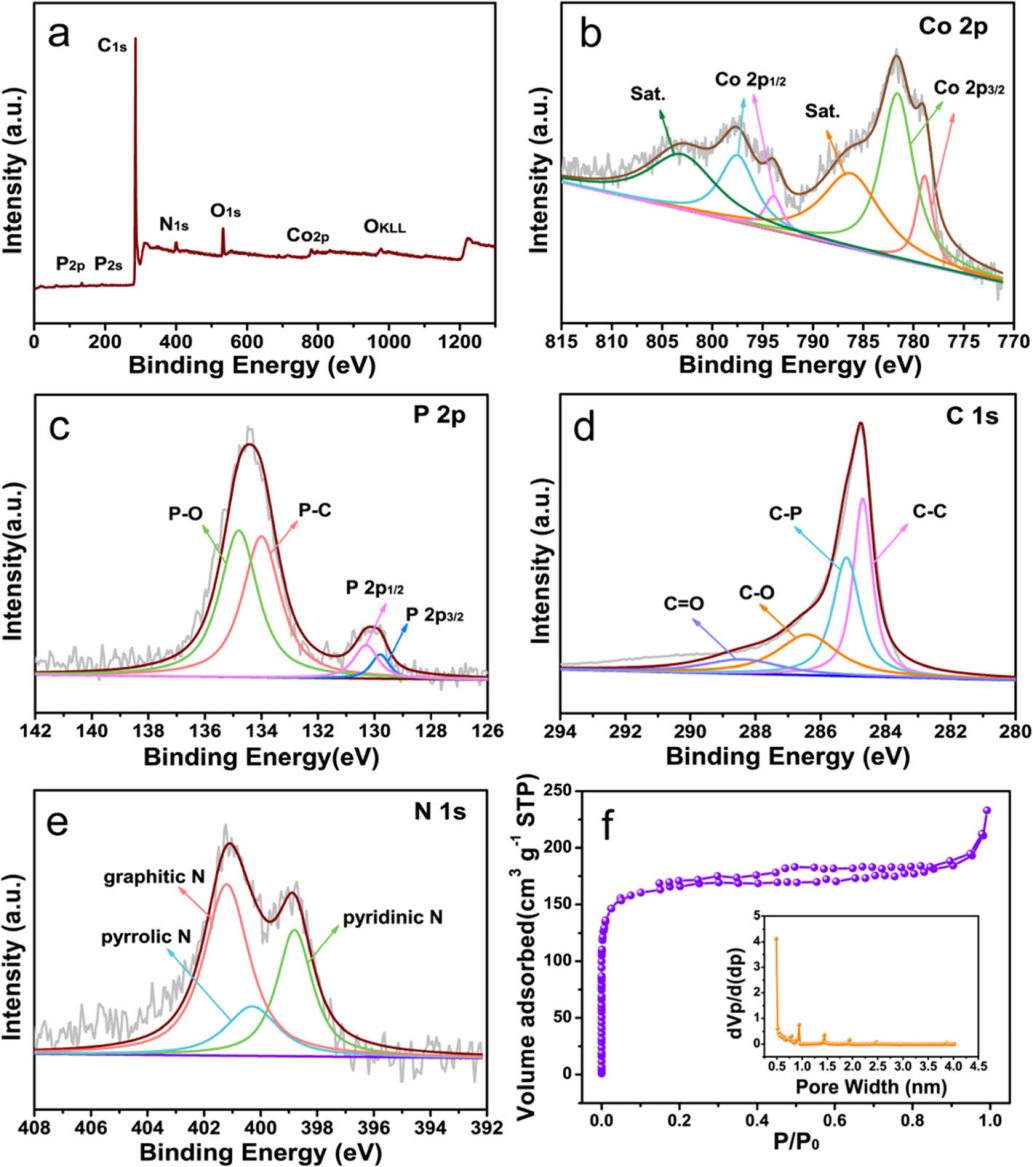
**a** XPS spectrum of NPMCNT-300 electrocatalyst. **b** Co 2p XPS spectrum of NPMCNT-300 electrocatalyst. **c** P 2p XPS spectrum of NPMCNT-300 electrocatalyst. **d** C1 s XPS spectrum of NPMCNT-300 electrocatalyst. **e** N1 s XPS spectrum of NPMCNT-300 electrocatalyst. **f** N_2_ adsorption–desorption isotherms and the corresponding pore size distribution curves

The species and elemental composition of the NPMCNT-300 electrocatalyst were determined by XPS, Fig. 3 a displays the existence of Co, P, N, C, and O elements in the survey spectrum. The Co 2p spectrum in Fig. 3b shows peaks centered on 778.6 and 781.6 eV connected to Co 2p_3/2_, 793.9, and 797.5 eV are attributed to Co 2p_1/2_, respectively. The peaks centralized at 778.9 eV and 793.9 eV are associated with Co^3+^, other peaks were centered at 781.6 eV and 797.5 eV are connected to Co^2+^. In addition, the strong satellite peaks centered on 786.2 and 803 eV are attributed to the vibration of Co^3+^ [21, 49–51]. As shown in Fig. 3c in the P 2p spectrum, the band of 129.8 eV is connected to P 2p_3/2_, while the band of 130.3 eV corresponds to P 2p_1/2_. Two peaks of 129.8 and 130.3 eV are correlated to CoP. Another peak at 134.0 eV is attributed to P–C, while the peak located at 134.8 eV is associated with P–O [41, 52, 53]. These results confirmed that NaH_2_PO_2_ acts as a phosphorus source for doping into CNTs and forming CoP. In Fig. 3d, the C1 s spectrum divided into four peaks (284.7, 285.2, 286.4, and 288.4 eV). The strong peak centralized at 284.7 eV corresponding with sp^2^ C = C energy of pyrolytic graphite. The peak (285.2 eV) is associated with the C–P matrix to the sp^2^ C bonded to P in the aromatic ring. Moreover, the peak of 286.4 eV is assigned to the C–O band. In addition, the peak of 288.4 eV is associated with C = O [30, 50, 54, 55]. The high-resolution N1 s peak of NPMCNT-300 is shown in Fig. 3e and it can be fitted by three peaks located at 398.8, 400.3, and 401.2 eV identifying with pyridinic N, pyrrolic N, graphitic N, respectively [56, 57]. The above XPS results demonstrate that P and N are doped into the defect sites of the CNTs by replacing the O or C atoms.

### Brunauer–Emmett–Teller (BET) Characterization

The nitrogen adsorption/desorption isotherms of NPMCNT-300 is shown in Fig. 3f. It is worth mentioning that the isotherms show a type-IV hysteretic loop, which demonstrates the presence of numerous mesoporous/microporous in NPMCNT-300 [58, 59]. And the BET surface area value of NPMCNT-300 electrocatalyst is 641 m^2^ g^−1^, these consequences show that the presence of nanotubes during the synthesis of NPMCNT-300 can greatly increase the specific surface area and pore volume of the electrocatalyst. This unique porous structure with large specific surface area is thought to be important for oxygen absorption and transportation of reactant molecules and exposure of the most active substances.

### Electrocatalytic Performance and Discussion

**Fig. 4:**
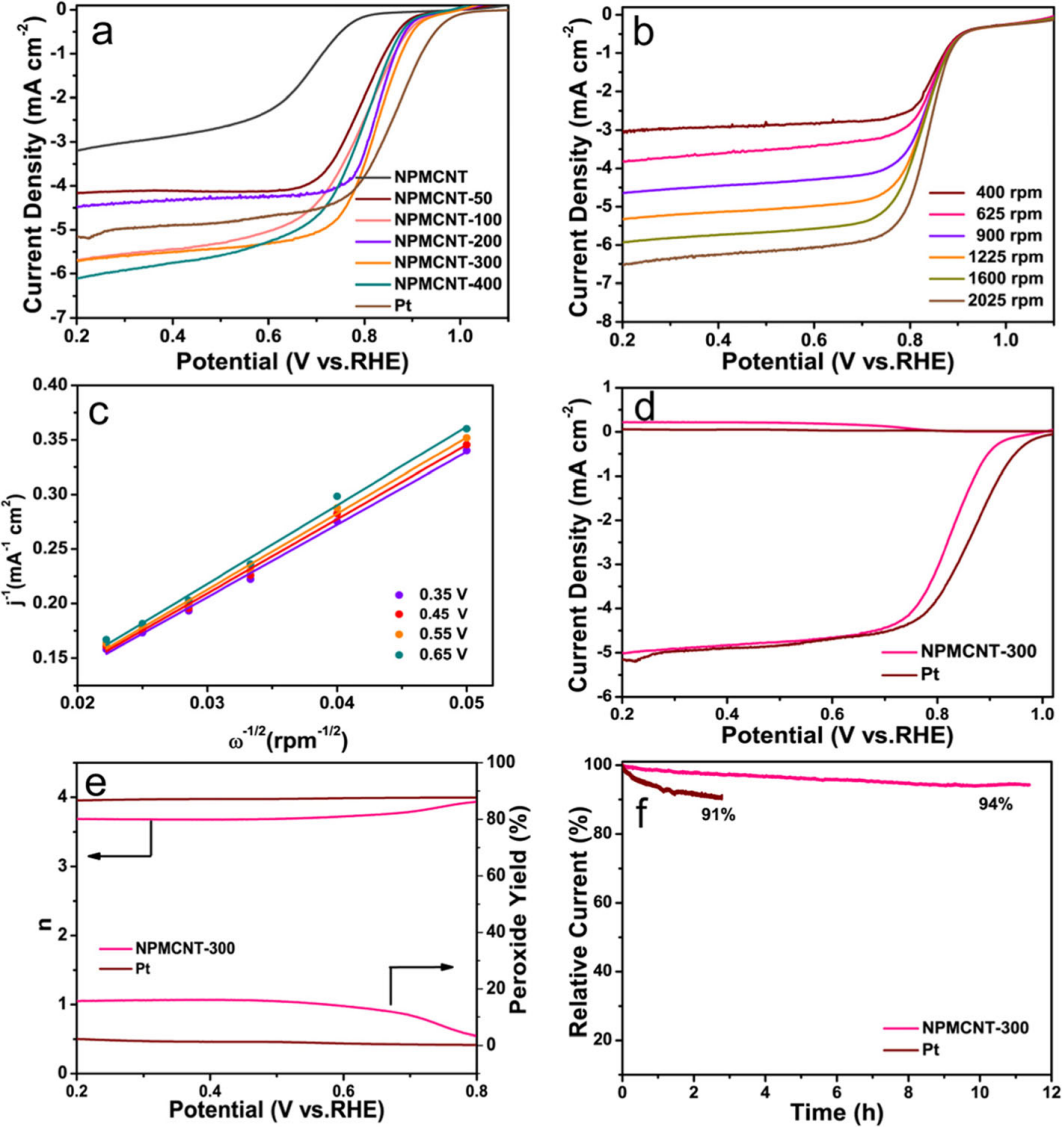
**a** Linear sweep voltammetry curves of the NPMCNT, NPMCNT-50, NPMCNT-100, NPMCNT-200, NPMCNT-300, NPMCNT-400, and 20% Pt/C electrocatalysts. **b** The rotating disk electrode voltammograms of NPMCNT-300 electrocatalyst with different rotation speeds. **c** The Koutecky-Levich plots (*j* is the measured current density, *ω* is the angular velocity of the disk (*ω* = 2π*N*, *N* is the linear rotation speed), **d** The rotating ring-disk electrode voltammograms, **e** the estimated value of electron transfer (*n*) and peroxide yields, and **f** the durability measurement of the NPMCNT-300 and Pt/C electrocatalysts

The electrocatalytic activity was tested using a three-electrode system for ORR. In Fig. 4a, the LSV curves were examined in an O_2_-saturated electrolyte. The onset potentials of NPMCNT, NPMCNT-50, NPMCNT-100, NPMCNT-200, NPMCNT-300, and NPMCNT-400 are 0.80, 0.89, 0.91, 0.90, 0.93, and 0.89 V (vs. RHE), respectively. Clearly, NPMCNT-300 exhibits the highest electrocatalytic activity. Compared with 40% Pt/C (0.993 V vs. RHE ), the performance of former is slightly weaker, however, diffusion-limited current density for NPMCNT-300s is close to 6 mA cm^−2^, which is better than Pt/C (5.1 mA cm^−2^). Figure 4 b shows typical LSV curves for NPMCNT-300 at different various rotational speeds (from 625 to 2025 rpm). The value of electron transfer number for ORR process of NPMCNT-300 is calculated to be close to 4 when the potential is from 0.35 to 0.65 V, which confirms the four-electron transfer pathway (Fig. 4c). To estimate the ORR kinetics, the number of electron transfer and yield of H_2_O_2_ and were measured by RRDE method. The corresponding ring current is contemporaneously measured with a Pt ring electrode for detection of peroxide species at the disk electrode (Fig. 4d). The number of electron transfer (Fig. 4e) of the NPMCNT-300 was about 3.7, which is agree well with the reckoned data from K–L equation, indicating that the ORR process follows an efficient four-electron approach. In the presence of these electrocatalysts, the intermediate H_2_O_2_ formation rate is low, which is about 17%. In order to measure the stability of the electrocatalyst, we used the i-t method to characterize the electrocatalyst at a voltage of 0.5 V and the rotation speed of 1600 rpm in O_2_-saturated 0.1 M KOH electrolyte. Figure 4 f shows the relative current density. After 40,000 s of continuous operation, NPMCNT-300 maintains a high relative current density of 94%, whereas, the initial current density was retained only 91% for Pt/C after continuous operation for 10,000 s, which indicates that the stability of NPMCNT-300 electrocatalyst is superior to the 40% Pt/C electrode.

**Fig. 5:**
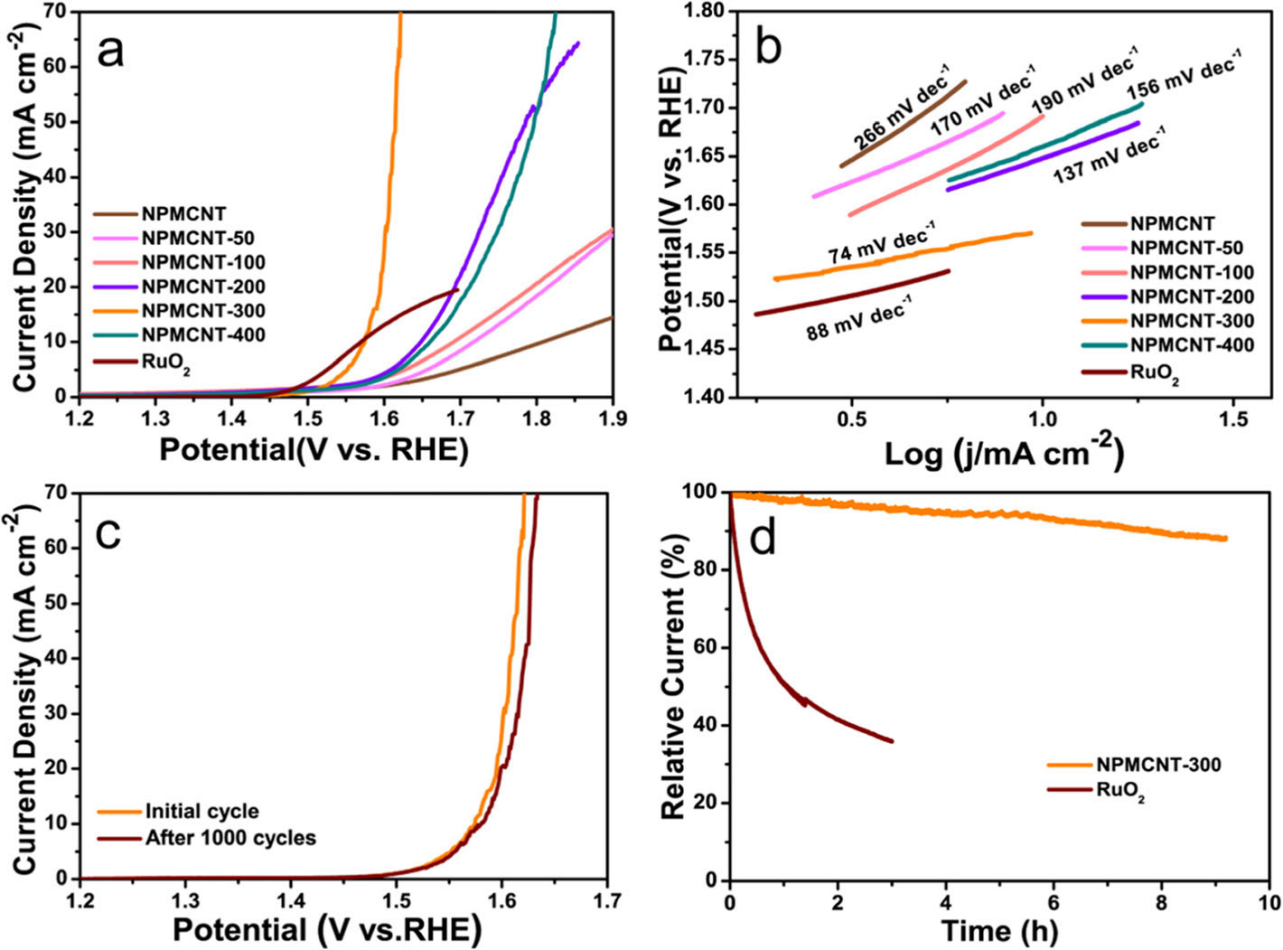
**a** Linear sweep voltammetry curves of electrocatalysts with iR compensation. **b** Tafel plots of electrocatalysts calculating from Fig. **a**. **c** Linear sweep voltammetry curves for initial and after 1000 cycles cyclic voltammetry. **d** Amperometric i-t curves

To assess the electrocatalytic performance for OER of the NPMCNT-300, the LSV curves were tested at the scanning rate of 5 mV s^−1^. In Fig. 5a, NPMCNT-300 electrocatalyst exhibits overpotential of 342 mV, which is equivalent to the potential of RuO_2_ electrocatalyst (340 mV). While for NPMCNT, NPMCNT-50, NPMCNT-100, NPMCNT-200, and NPMCNT-400 were 579, 488, 461, 418, and 430 mV, respectively. Figure 5 b shows that the Tafel slope of NPMCNT-300 electrocatalyst is 74 mV dec^−1^ and for NPMCNT, NPMCNT-50, NPMCNT-100, NPMCNT-200, and NPMCNT-400 are 266, 170, 190, 137, 156 mV dec^−1^, respectively. While the NPMCNT-300 electrocatalyst is lower than RuO_2_ (88 mV dec^−1^), therefore proving the excellent OER kinetics of NPMCNT-300 electrocatalyst. This results show that NPMCNT-300 has the excellent electrocatalytic performance as RuO_2_ for OER. In order to investigate the durability of the NPMCNT-300 electrocatalyst, two methods were used. First, NPMCNT-300 was tested in a KOH electrolyte for 1000-cycle CV (Fig. 5c). After the test, it showed a little reduction in degradation (5 mV). Another stability test was using the chronoamperometry method. The chronoamperometry method is to record the change of current with time by applying a large step potential (from a potential jump occurring in a Faraday reaction to an effective potential approaching zero of the surface electroactive component of the electrode) to the working electrode in the unstirred solution. The initial potential was based on the results from Fig. 5d, which makes the NPMCNT-300 and RuO_2_ to produced 10 mA cm^−2^ within iR compensation. The current of NPMCNT-300 electrocatalyst is retained for about 90% for 9 consecutive hours, while RuO_2_ loses more than 50% of the current only in 1 h. Both stability tests indicate NPMCNT-300 has excellent stability for OER. Comparison of the electrocatalytic performance of CoP with various reported Co-based non-precious electrocatalysts in alkaline media in Table 1.

**Table 1:** Comparison of the electrocatalytic performance of CoP with various reported Co-based non-precious electrocatalysts in alkaline media

Electrocatalyst	OER (η (mV) at *j* = 10 mA cm^−2^)	ORR (E_1/2_ V)	Reference
Co_2_P@NPC-1	327	0.83	[2]
NiP@N,P-CNSs	390	0.75	[7]
Co@N-CNTF	350	0.81	[11]
CoP NPs/CNSs	340	0.88	[60]
CoP-PBSCF	378	0.75	[61]
Bi–CoP/NP-DG	370	0.81	[62]
CoP_x_/CoN_x_C@CNT	460	0.83	[63]
CoNP@NC/NG-700	390	0.83	[64]
CoZn-NC-800	480	0.82	[65]
Co-N/C-800	492	0.67	[66]
Co/N-GCA	408	0.81	[67]
**Co/CoP**	**342**	**0.93**	**This work**

The above results summarize the corresponding OER and ORR electrochemical performances of different products, indicating that the intake of different phosphorus sources will affect the performance of the electrocatalysts. On one hand, although the electrocatalysts of NPMCNT-50, NPMCNT-100, and NPMCNT-200 have similar structures, the phosphorus content is lower which will cause the amount of CoP formation is less. On the other hand, although NPMCNT-400 contains the highest phosphorus content, due to the destruction of the original CNT structure, the CNTs clumped together and the electrocatalytic activity was relatively poor. The special morphology of NPMCNT-300 provides larger specific surface area as well as higher amount of CoP, resulting in improved electrochemical performance.

**Fig. 6:**
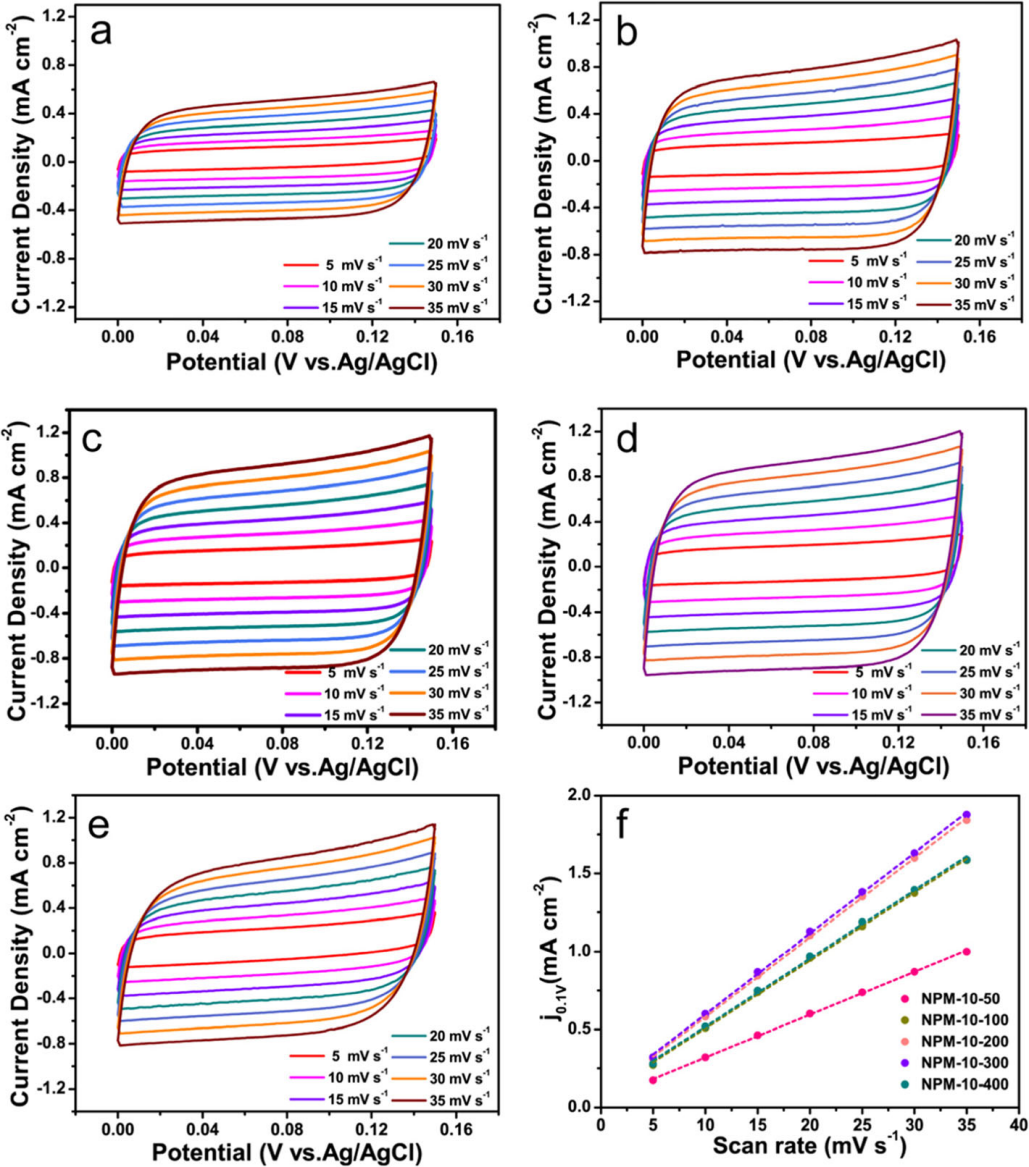
Cyclic voltammetry scans of **a** NPMCNT-50, **b** NPMCNT-100, **c** NPMCNT-200, **d** NPMCNT-300, and **e** NPMCNT-400. **f** Plots between current density and scan rate for the electrocatalysts

The electrochemically active surface area (ECSA) of the electrocatalysts can further indicate the cause of the excellent electrochemical activity. The double-layer capacitance (C_dl_) of NPMCNT-50, NPMCNT-100, NPMCNT-200 NPMCNT-300, and NPMCNT-400 was calculated at different various scan rates (0.005, 0.01, 0.15, 0.20, 0.25, 0.30, and 0.35 V s^−1^) in Fig. 6a–e. In order to measure the electrochemical double-layer charge by CV, a potential range in which no significant Faraday process occurs is determined from the static CV. This range is typically a 0.1 V potential window centered at the open-circuit potential (OCP) of the system. All currents measured in this non-Faraday potential region are considered to be due to double-layer charging. Figure 6 f displays the plots between the various scan rates and the current density of the electrocatalyst at 0.1 V (vs*.* Ag/AgCl). The double-layer charging current is equal to the product of the scan rate, *v*, and the electrochemical double-layer capacitance, *C*_dl_, as given by Eq. (1):

13 $$ ic=v\ {C}_{\mathrm{dl}} $$

Thus, a plot of *ic* as a function of *v* yields a straight line with a slope equal to *C*_dl_. By plotting the Δj against the scan rate at 0.1 V (vs. Ag/AgCl), the slope which is twice of *C*_dl_ can be obtained as shown in Fig. 6f. The *C*_dl_ of linear fitting slope are 27.55, 43.55, 51, 51.75, and 43.73 mF cm^−2^ for NPMCNT-50, NPMCNT-100, NPMCNT-200, NPMCNT-300, and NPMCNT-400, respectively. The ECSA of a electrocatalyst sample is calculated from the C_dl_ according to Eq. (2):

14 $$ \mathrm{ECSA}={C}_{\mathrm{dl}}/{C}_{\mathrm{s}} $$

where *C*s is the specific capacitance of the sample or the capacitance of an atomically smooth planar surface of the material per unit area under identical electrolyte conditions. By considering the specific capacitance of an atomically smooth planar surface with a real surface area of 1.0 cm^2^, the specific capacitance (*C*s) is generally within 20–60 μF cm^−2^ in alkaline media. For our estimates of surface area, we use general specific capacitances of Cs = 0.04 mF cm^−2^ in 0.1 M KOH. From this, we estimate that the ECSA are 0.0689, 0.1089, 0.1275, 0.1294, and 0.1093 m^2^ for NPMCNT-50, NPMCNT-100, NPMCNT-200, NPMCNT-300, and NPMCNT-400 electrocatalysts. Therefore, the NPMCNT-300 electrocatalyst exhibits excellent performance for OER and ORR.

## Conclusions

We make full use of the effective specific surface area of MOFs and high activity of CoP to produce excellent bifunctional electrocatalyst. The uniform introduction of cobalt sources on the surface of MOFs nanosheets by atomic layer deposition (ALD) techniques, and the derivation of N-doped nanotubes during high-temperature calcination, and encapsulation of Co/CoP in the tip of the nanotubes were reported. It is confirmed that the presence of nanotubes provides a larger specific surface area for the electrocatalyst. When used as a bifunctional electrocatalyst, NPMCNT-300 exhibits extraordinary electrochemical performance for both OER and ORR. It was demonstrating an onset-potential of 0.925 V for ORR and the overpotential is about 342 mV with a Tafel slope of 74 mV dec^−1^ for OER. Moreover, the electrocatalyst displayed prominent stability for both OER and ORR.

## Data Availability

The datasets generated during and/or analyzed during the current study are available from the corresponding author on reasonable request.

## Abbreviations

OER: Oxygen evolution reaction
ORR: Oxygen reduction reaction
TMPs: Transition metal phosphides
CoP: Cobalt phosphide
MOFs: Metal-organic frameworks
CNTs: Carbon nanotubes
NPM: N-doped porous MOFs nanosheets
PE-ALD: Plasma-enhanced atomic layer deposition
XRD: X-ray diffraction
FESEM: Field emission scanning electron microscope
TEM: Transmission electron microscope
EDS: Energy dispersive X-ray spectroscopy
XPS: X-ray photoelectron spectroscopy
RRDE: Rotating ring-disk electrode
LSV: Linear sweep voltammogram
RHE: Reversible hydrogen electrode
K-L: Koutecky-Levich
ECSA: Electrochemical active surface area
CV: Cyclic voltammetry
C_dl_: Double-layer capacitance
